# Supplementary material for: Group size effect on cooperation in one-shot social dilemmas
Source: Sci Rep. 2015 Jan 21;5:7937. doi: 10.1038/srep07937 (PMC4300455; doi:10.1038/srep07937)
Supplement: Supplementary Information — Proofs of the theoretical results [file srep07937-s1.pdf]

Supplementary Information for 'Group size effect on cooperation in one-shot social dilemmas'

Hélène Barcelo[1] & Valerio Capraro[2]

[1]*Mathematical Sciences Research Institute, CA 94720, Berkeley, USA*
[2]*Center for Mathematics and Computer Science (CWI), 1098 XG, Amsterdam, The Netherlands.*

In this Supplementary Information we collect the proofs of all the results we stated in the Theoretical Results section.

Proof of Proposition 1. Fix, for notational simplicity, $i = 1$. We have $U_1(C, \dots, C) = b - c$ and

$$\begin{aligned} U_1(D, C, \dots, C) &= b - \beta_1 \left(b - b\frac{N-2}{N-1} + c \right) \\ &= b - \frac{b\beta_1}{(N-1)} - \frac{c\beta_1}{N-1}. \end{aligned}$$

It is clear that if N is large enough (depending on the player and on b and c), one has $U_1(D, C, \dots, C) > U_1(C, \dots, C)$. □

Proof of Proposition 2. Fix player $i = 1$. We have $U_1(1, \dots, 1) = \gamma N$ and

$$\begin{aligned} U_1(0, 1, \dots, 1) &= \gamma(N-1) + 1 - \beta_1 (\gamma(N-1) + 1 - \gamma(N-1)) \\ &= \gamma(N-1) + 1 - \beta_1. \end{aligned}$$

It is clear then clear that the condition $U_1(0, 1, \dots, 1) > U_1(1, \dots, 1)$ is independent of N. □

Proof of Proposition 3. Fix $i = 1$. We have $U_1(C, \dots, C) = \alpha_1(b - c) + (1 - \alpha_1)(b - c)(N - 1)$ and

$$\begin{aligned} U_1(D, C, \dots, C) &= \alpha_1 b + (1 - \alpha_1) b + (1 - \alpha_1)(N-1) \left(\frac{b(N-2)}{N-1} - c \right) \\ &= b + (1 - \alpha_1)(bN - 2b - cN + c). \end{aligned}$$

Observe that the condition $U_1(D, C, \dots C) > U_1(C, \dots, C)$ reduces to $\alpha > 1 - \frac{c}{b}$ and so it does not depend on N. □

Proof of Proposition 4. We have $U_1(1,\dots,1) = \alpha_1\gamma N + \gamma N^2 - \alpha_1\gamma N^2$ and

$$\begin{aligned} U_1(0,1,\dots,1) &= \alpha_1(1+\gamma(N-1)) + (1-\alpha_1)(1+\gamma(N-1)) + \gamma(1-\alpha_1)(N-1)^2 \\ &= 1 - \gamma N + \gamma N^2 + 2\alpha_1\gamma N - \alpha_1\gamma N^2 - \alpha_1\gamma. \end{aligned}$$

It is then clear that the condition $U_1(0,1,\dots,1) < U_1(1,\dots,1)$ reduces to

$$1 - \alpha_1\gamma + \gamma N(\alpha_1 - 1) < 0,$$

which is always verified if N is large enough. □

Proof of Theorem 1. Since $\mathcal{G}_{p_s} = \mathcal{G}$, then σ^{p_s} is the Nash equilibrium of the original game. Since there is no incentive to deviate from a Nash equilibrium, the τ measure is the Dirac measure concentrated on $J = \emptyset$. Therefore $v_i(p_s)$ coincides with the payoff in equilibrium; that is, $v_i(p_s) = 1$.

Let now p_c be the fully cooperative coalition structure and observe that, for all $j \in P$, one has $I_j(p_c) = 1 - \gamma$ and $D_j(p_c) = \gamma N - 1$. Consequently, $\tau_{i,j}(p_c) = \frac{1-\gamma}{\gamma(N-1)}$, for all $i, j \in P$, $i \neq j$. Now, $e_{i,J}(p_c) = \gamma$, for all $J \neq \emptyset$, and $e_{i,\emptyset}(p_c) = \gamma N$. Therefore,

$$\begin{aligned} v_i(p_c) &= \gamma N \left(1 - \frac{1-\gamma}{\gamma(N-1)}\right)^{N-1} + \gamma\left(1 - \left(1 - \frac{1-\gamma}{\gamma(N-1)}\right)^{N-1}\right) \\ &= \gamma N \left(\frac{\gamma N - 1}{\gamma(N-1)}\right)^{N-1} + \gamma\left(1 - \left(\frac{\gamma N - 1}{\gamma(N-1)}\right)^{N-1}\right). \end{aligned}$$

To compute the cooperative equilibrium, we observe that this would be the lowest contribution among the ones which, if contributed by all players, would give to all players a payoff of at least $v_i(p_c)$. To compute this contribution it is enough to solve the equation

$$1 - \lambda + \gamma N\lambda = v_i(p_c),$$

whose solution is indeed $\lambda = \frac{v_i(p_c)-1}{\gamma N - 1}$, as stated.

It remains to show that the cooperative equilibrium is increasing with N. To this end, we replace N by a continuous variable $x \geq 2$ and denote $v(x) := v_i(p_c)$, $f(x) = \frac{v(x)-1}{\gamma x - 1}$, and $r(x) = \left(\frac{\gamma x - 1}{\gamma(x-1)}\right)^{x-1}$. Observe that all these functions are differentiable in our domain of interest $x \geq 2$. Our aim is to show that $f(x)$ is increasing, that is, $f'(x) > 0$. We start by observing that $r(x)$ is increasing. This can be seen essentially in the same way as one sees the standard fact that $\left(1 + \frac{1}{n}\right)^n$ is increasing in n, by using Bernoulli's inequality. Hence, we have

$$v'(x) = \gamma r(x) + \gamma r'(x)(x-1) > \gamma r(x).$$

Consequently, using also the fact that $\gamma x r(x) = v(x) - \gamma(1 - r(x))$, we conclude

$$\begin{aligned} f'(x) &= \frac{v'(x)(\gamma x - 1) - \gamma(v(x) - 1)}{(\gamma x - 1)^2} \\ &> \frac{\gamma(\gamma x r(x) - v(x) + 1)}{(\gamma x - 1)^2} \\ &= \frac{\gamma(1 - \gamma(1 - r(x)))}{(\gamma x - 1)^2} \\ &> 0, \end{aligned}$$

where, the last inequality follows from the fact that both γ and $r(x)$ are strictly smaller than 1. □

Proof. The forecast associated to the selfish coalition structure is $v_i(p_s) = 0$, for all players, corresponding to the payoff in (Nash) equilibrium. To compute the forecast associated to the fully cooperative coalition structure, observe that $e_{i,\emptyset}(p_c) = b - c$, corresponding to Pareto optimum where all players cooperate. The incentive to deviate from the cooperative strategy is $I_j(p_c) = c$, while the disincentive is $D_j(p_c) = b - c$, corresponding to the loss incurred in case all other players anticipate player j's defection and decide to defect as well. Finally, $e_{i,J}(p_c) = -c$, for all $J \neq \emptyset$, corresponding to the strategy profile where only player i cooperates and all other players defect. Hence we have

$$v_i(p_c) = (b - c)\left(1 - \frac{c}{b}\right)^{N-1} - c\left(1 - \left(1 - \frac{c}{b}\right)^{N-1}\right).$$

Of course, if $v_i(p_c) \leq 0$, then the cooperative equilibrium coincides with the Nash equilibrium. Otherwise, by symmetry, it is the only strategy σ such that

$$u_i(\sigma, \dots, \sigma) = v_i(p_c), \tag{1}$$

for all $i \in P$. Setting $\sigma = \lambda C + (1 - \lambda)D$, we obtain

$$\begin{aligned} u_i(\sigma, \dots, \sigma) &= \lambda \sum_{k=0}^{N-1} \lambda^{N-1-k}(1-\lambda)^k \binom{N-1}{k} \left(\frac{b(N-1-k)}{N-1} - c\right) \\ &\quad + (1-\lambda) \sum_{k=0}^{N-1} \lambda^{N-1-k}(1-\lambda)^k \binom{N-1}{k} \left(\frac{b(N-1-k)}{N-1}\right) \\ &= \sum_{k=0}^{N-1} \lambda^{N-1-k}(1-\lambda)^k \binom{N-1}{k} \frac{b(N-1-k)}{N-1} - c\lambda \\ &= b - c\lambda - \frac{b}{N-1} \sum_{k=0}^{N-1} \lambda^{N-1-k}(1-\lambda)^k \binom{N-1}{k} k. \end{aligned}$$

Now we use the fact that

$$\sum_{k=0}^{N-1} \lambda^{N-1-k}(1-\lambda)^k \binom{N-1}{k} k = (1-\lambda)(N-1),$$

to reduce Equation (1) to

$$\lambda(b-c) = v_i(p_c), \tag{2}$$

which concludes the proof. $\square$
